# What impact does oocyte vitrification have on epigenetics and gene expression?

**DOI:** 10.1186/s13148-020-00911-8

**Published:** 2020-08-10

**Authors:** Julie Barberet, Fatima Barry, Cécile Choux, Magali Guilleman, Sara Karoui, Raymond Simonot, Céline Bruno, Patricia Fauque

**Affiliations:** 1 CHU Dijon Bourgogne, Laboratoire de Biologie de la Reproduction, CECOS, 14 rue Gaffarel, 21079 Dijon Cedex, France; 2 Gynécologie-Obstétrique, CHU Dijon Bourgogne, 14 rue Gaffarel, 21079 Dijon Cedex, France

**Keywords:** DNA methylation, Epigenetics, Gene expression, Oocyte, Vitrification

## Abstract

Children conceived by assisted reproductive technologies (ART) have a moderate risk for a number of adverse events and conditions. The question whether this additional risk is associated with specific procedures used in ART or whether it is related to the intrinsic biological factors associated with infertility remains unresolved. One of the main hypotheses is that laboratory procedures could have an effect on the epigenome of gametes and embryos. This suspicion is linked to the fact that ART procedures occur precisely during the period when there are major changes in the organization of the epigenome. Oocyte freezing protocols are generally considered safe; however, some evidence suggests that vitrification may be associated with modifications of the epigenetic marks. In this manuscript, after describing the main changes that occur during epigenetic reprogramming, we will provide current information regarding the impact of oocyte vitrification on epigenetic regulation and the consequences on gene expression, both in animals and humans. Overall, the literature suggests that epigenetic and transcriptomic profiles are sensitive to the stress induced by oocyte vitrification, and it also underlines the need to improve our knowledge in this field.

## Background

Since their introduction, medically assisted reproductive technologies (ARTs) have allowed millions of children to be born to infertile couples, accounting for 2 to 6% of births in Europe [1, 2]. Although generally recognized as safe, associations exist between ARTs and an increased incidence of low birth weight, birth defects, growth and metabolic disorders, and psychomotor or mental developmental delays [3]. More specifically, there has been an increase in the occurrence of rare diseases related to genomic imprinting, such as Beckwith-Widemann syndrome, Angelman syndrome, and Silver-Russell syndrome [4]. ART could be detrimental to epigenetic reprogramming of gametes and pre-implantation embryos, leading to potential effects after birth [5]. The periconception period—gametogenesis, fertilization, and early embryonic development—is a time of physiologically intense epigenetic reprogramming [6, 7].

Cryopreservation of oocytes by “slow freezing” was initiated in the 1980s, and the first birth was obtained in Japan more than 30 years ago [8]. However, one of the main difficulties with this technique is that oocyte survival rates have remained low, around 60% post-thaw [9]. The appearance of oocyte vitrification has subsequently completely revolutionized this field. Many studies have proven that oocyte survival rates, fertilization rates, and embryonic cleavage were higher after vitrification than after slow freezing, and the results with vitrification may even be equivalent to the results obtained with fresh oocytes [10–18].

In view of the growing number of children conceived thanks to oocyte vitrification and the absence of birth defects in these children [19–21], it is now considered by many countries as the first-line technique to preserve female fertility [22]. Obviously, it is used in women who need potentially sterilizing treatment or whose fertility may be prematurely impaired. Oocyte vitrification is also on the rise in the context of intra-couple ART management; it is often used in the event of failed sperm collection or when there is a preference for the storage of oocytes rather than embryos [23]. In addition, it could be chosen in the context of oocyte donation program, but also in some countries where the law prohibits embryo cryopreservation. Finally, some countries have expanded the use of oocyte freezing for purpose of postponing pregnancy for personal or professional reasons, also known as “delayed childbearing” [24].

It is therefore important that the technique used for freezing oocytes is not harmful for future pregnancies or the health of children that may be obtained from these oocytes. However, pregnancy outcomes from vitrified oocytes vary between centres, who use different techniques and whose operators have different levels of expertise [14]. There is usually a shallow learning curve for oocyte vitrification techniques, resulting in a maximum survival rate of no more than 70–80% [14]. Furthermore, it has been reported in mammals that oocyte vitrification could reduce the potential for embryonic development [25–27]. The effects of vitrification can be at the molecular level and may influence epigenetic control, especially since these processes coincide with the reprogramming of the oocyte epigenome. Besides, the challenge for the oocyte is the synthesis and preservation of all transcripts (in particular expression of epigenetic modifier enzymes) needed to fulfil protein requirements during the period of meiotic completion, fertilisation, and the oocyte-embryo transition [28]. During oogenesis, the transcription influenced by epigenetic factors is intense. The growth phase is succeeded by a phase of resumed meiotic activity and transcriptional silencing [28] in which changes in gene expression depend on translation and degradation of transcripts. This is crucial for assembling the molecular machinery, in particular, the transcripts involved in epigenetic mechanisms, required for meiotic progression, fertilization, and embryo development [29, 30]. The transcripts content shows that temporal changes are mostly regulated via epigenetic mechanisms and associated with oocyte competencies [31]. Therefore, any factors, herein potentially linked to the vitrification procedures (consequences of chilling and high concentrations of cryoprotectants), that influence genome integrity [32] and transcripts synthesis/repression, stability, and association with the translation machinery can have a major impact on protein expression and crucial biological processes.

In this manuscript, after describing (1) the epigenetic marks controlling gene transcription as well as the major epigenetic events that occur during gametogenesis and (2) gene expression regulation through small RNAs, we will discuss the current state of knowledge of the impact of oocyte vitrification on epigenetic regulation and the consequences on gene expression.

## Epigenetic reprogramming during oogenesis

Epigenetics refers to the processes leading to the diversification of the expression of genetic material in a heritable manner during cell divisions and without modifying the nucleotide sequences. These mechanisms, which occur during development, are numerous and complex and directly or indirectly influence the state of chromatin. DNA methylation appears as a major epigenetic mark involving a covalent modification of DNA. Indeed, DNA methylation is a biochemical process where a methyl group from the donor S-adenosyl methionine is added at the carbon-5 position of cytosine (5mC) in the cytosine-guanine (CpG) dinucleotide by DNA methyltransferases (DNMTs) and can be erased by 5mC oxydases (ten-eleven translocation proteins: TET), through sequential oxidation of 5mC to 5-hydroxymethylcytosine (5hmC), 5-formylcytosine (5fC), and finally, 5-carboxylcytosine (5caC) [33, 34]. Within the context of chromatin, DNA methylation does not function alone. Instead, there is a complex interplay between DNA methylation and post-translational biochemical histone modifications (mainly localized in the *N*-terminal tails) known as the “histone code” [35]. Together, they control chromatin accessibility and packaging, resulting in gene activation or repression.

Epigenetic marks are also involved in the fine regulation of a small group of genes called “imprinted genes” (about 150 described in mice, and about half of these genes have been found in humans [36, 37]). These genes have a monoallelic expression that is dependent on the parental origin of the allele. The parental imprint is linked to differential epigenetic labeling of parental alleles, established during gametogenesis. Mature gametes then transmit their own parental epigenome, which is maintained during successive cell divisions after fertilization. This phenomenon affects different regions and plays a major role in the development and growth of the conceptus.

Beyond being essential for the acquisition and maintenance of gametic identity, pluripotency embryonic lineage decisions, and genomic imprinting, reprogramming of the epigenome is crucial for the control of repeated sequences, especially transposable elements (TEs) [38, 39]. TEs represent more than half of the human genome [40, 41]. Some of these elements, retrotransposons in particular, have retained some level of activity (even in the human embryo, as reported by Grow et al.) [42] and the ability to move [43]. Consequently, they can have an impact on gene organization and expression, for instance, initiating chromosomal rearrangements and gene mutations/deletion/duplication or inappropriate gene expression [38, 44, 45]. Indeed, experimental overexpression of on type of transposable element (a non-long terminal repeats [LTRs] sub-type: LINE-1) in mouse oocytes results in oocyte aneuploidy and embryonic lethality [46]. Therefore, the organism has developed several defence mechanisms to silence some TEs, such as the methylation of DNA, histone modifications, and regulation by small RNAs [47].

More generally, the facts that some TEs are sensitive to environmental factors that can mediate their mobilization and that epigenetic modifications are also sensitive to the environment suggest that both can work together [47], underscore the importance to determine whether some reproductive procedures such as oocyte vitrification could perturb transposable element control during this critical window of development. Otherwise, in the oocyte, LTRs, another subtype of transposable elements, are highly expressed, very active, and can regulate host genes, notably by contributing to the generation of hypermethylated domains downstream [48–50].

### DNA methylation

In mammals, DNA methylation of CpG sites is generally high across gene bodies and inter-genic regions, with low or intermediate DNA methylation observed almost solely at regulatory regions, such as promoters and enhancers [51]. DNA methylation in CpGs sequence is indeed one of the main epigenetic mechanisms for the regulation of gene expression [52]. Certain regions of the genome contain clusters of CpG sequences (200–1000 bp in length), termed CpG islands (CGI), which are usually unmethylated and associated with gene transcription regulation [53, 54]. The CGI is mostly found in gene promoter regions in which DNA methylation can modify gene expression by regulating the recruitment of methylated DNA-binding proteins or by changes in the accessibility of the DNA sites, which influence transcription factor binding and overall chromatin structure [53, 54]. Commonly, DNA methylation of the promoter regions is generally associated with gene silencing, although this rule is not valid for promoters with low CpG density [55]. Furthermore, the methylation status of enhancers exhibiting widespread hypo-methylation during development [56], and for which interactions with promoters could be blocked by insulators, is also key to controlling gene expression in development and cell function [51, 57]. Therefore, DNA methylation is involved in many important epigenetic processes, such as mammalian development and cellular reprogramming and retrotransposon silencing, as reported above [58]. It is also one of the major mechanisms for initiating and maintaining parental imprinting. These imprinted genes are generally grouped into loci where each has regions that exhibit allele-specific differences in methylation, called differentially methylated regions (DMRs). Within these DMRs, imprinting control regions (ICRs) participate in the regulation of other genes that are subject to imprinting within the same locus (ICRs’ differential methylation patterns established in the gametes). Furthermore, CGI methylation during oocyte growth is not exclusively associated with genomic imprinting, but it determines a significant proportion of the genomic DNA methylation profile inherited by the preimplantation embryo [59, 60] beyond them are required to maintain transcriptional repression at a cluster of testis-specific genes or to repress brain-specific genes during embryonic development [60].

**Fig. 1:**
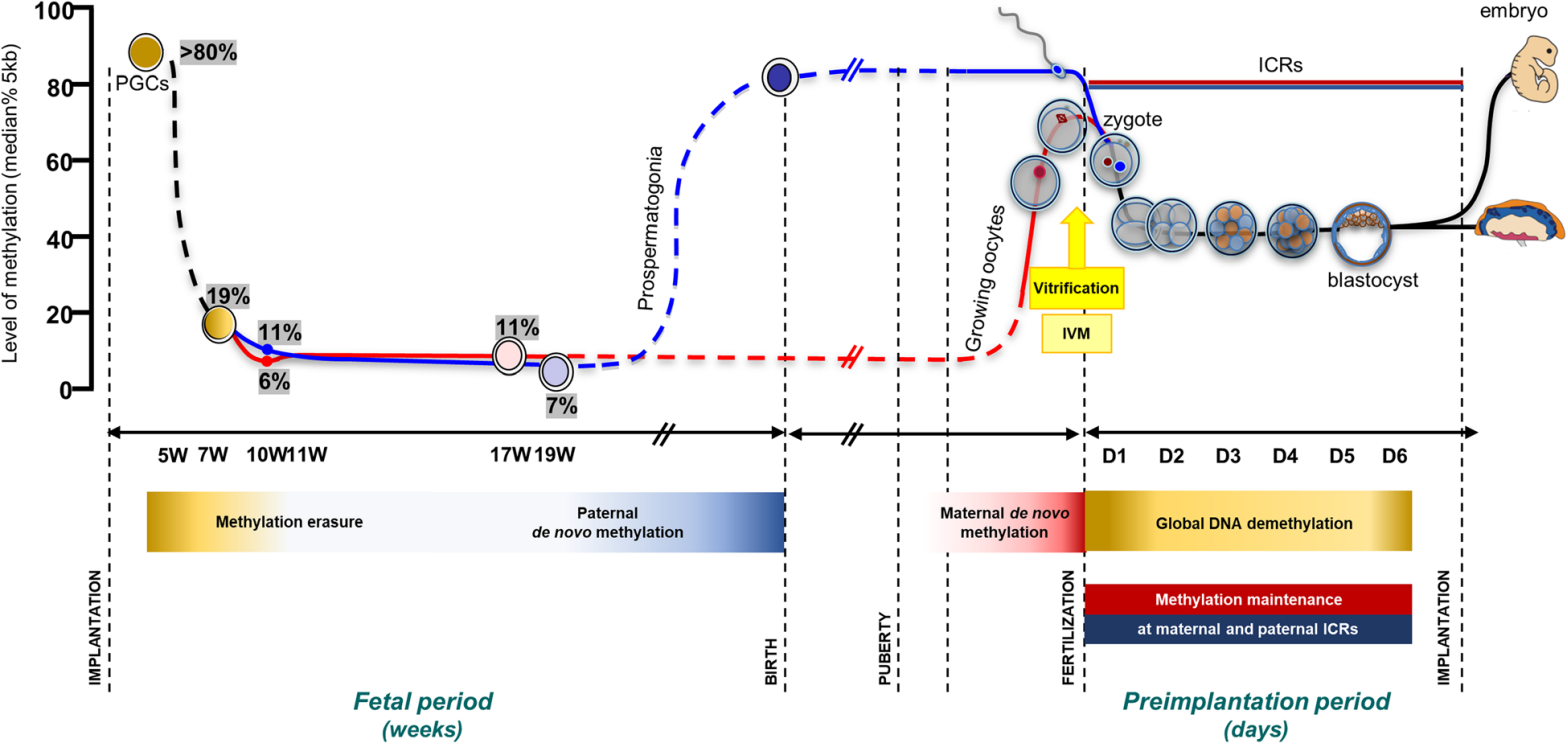
The timing of vitrification (associated or not with oocyte in vitro maturation) coincides with DNA methylation changes taking place during gametogenesis and embryogenesis. DNA methylation changes described in humans are represented here through full lines. The progenitors of the mouse and human germline (PGCs, Primordial Germ Cells) undergo a marked first genome-wide DNA demethylation. The gametic re-methylation will then be different between the two sexes. Indeed, on the male side, de novo methylation of germ cells is initiated and almost complete during prenatal development while on the female side, oocytes remain hypomethylated throughout the fetal period. After the puberty, DNA methylation is then acquired during the growing phase of the oocyte cohort. Following fertilization, maternal and paternal epigenomes introduced by the gametes must be reset a second time (second wave of demetylation) to establish the pluripotency that is required for development embryonic lineages. However, methylation of DNA acquired in the germ line at the ICRs will be maintained after fertilization to ensure sex-specific and monoallelic expression of imprinted genes

Thanks to new technologies which make it possible to analyze the epigenome from a few cells, major epigenetic events throughout the fetal and preimplantation periods (long based on data obtained in the mouse model) have been decoded in humans, particularly those related to DNA methylation [6, 61] (Fig. 1). In short, the primordial germ cells (PGC) will undergo a significant demethylation of their genome during the first weeks of development (levels are less than 10%) [62]. After the period of DNA demethylation, male germ cells initiate and complete remethylation during prenatal development until puberty through de novo DNA methyltransferase Dnmt3A and its cofactor, Dnmt3L [63–65]. In contrast, oocytes in the female arrested at the prophase of meiosis I remain hypomethylated throughout the fetal period [63–65]. DNA methylation will only be acquired by the same enzymatic machinery as that in male germ cells [66, 67] in the post-pubertal period during each cycle and in each cohort of oocytes engaged towards ovulation from the primary stage to the antral follicle stage [68]. The fact that this marking is gradually established throughout oocyte growth and that some methylation marks are not established until the final stages of the oocyte [59] draws attention to the potential deregulations induced by reproductive techniques, in particular, controlled ovarian hyperstimulation which aims to obtain several mature oocytes or in vitro oocyte maturation, and also potentially during the oocyte manipulations required in the oocyte freezing protocol [68]. It is interesting to note that ART children with Beckwith-Wiedemann syndrome all have methylation alterations carried by the maternal allele [69].

Following fertilization, the parental genome undergoes a massive second wave of demethylation (the paternal genome is actively demethylated, and the maternal genome passively demethylated [70]) to establish the pluripotency that is required to develop embryonic lineages. In contrast, the ICRs of imprinted genes escape demethylation thanks to the action of DNA methyltransferase 1 (Dnmt1) [71] and the KRAB zinc-finger protein 57 (ZFP57) protection. Oocyte ICRs are composed of CGIs enriched for a specific CG-rich hexanucleotide sequence (TGCCGC) recognised by the ZFP57 which protects imprinted sites against the wave of DNA demethylation during the embryonic reprogramming by recruiting KAP1 and other effectors [72, 73]. Thus, parental-specific DNA methylation of ICRs is acquired in the germline and must remain after fertilization.

The re-methylation of the genome then occurs in the cells of the inner cell mass of the blastocyst under the action of Dnmts [61]. All of the epigenetic modifications taking place in the embryo depend heavily on the level of epigenetic modifier enzymes expressed from maternally stored mRNAs during oocyte growth. One that is specifically inherited from the oocyte is Dnmt1o, an oocyte-derived isoform of Dnmt1 [74]. Dnmt1o, which is only expressed in oocytes and preimplantation embryos, is involved in the methylation maintenance of imprinted loci and is important for normal embryo development [75]. Disruptions in DNMT1o-dependent maintenance methylation have been suggested to explain the mosaic DNA hypomethylation at multiple imprinted loci associated with ART [76]. In humans, DNMT1o’s crucial role in maintaining methylation in early embryos has also been highlighted [77].

Furthermore, one of the major functions of DNA methylation is the silencing of retrotransposons in order to ensure normal meiosis and the preservation of genomic integrity in the oocyte [47]. However, some TEs could also be important in the contribution of intragenic DNA methylation in gametic DNA methylome. A recent study in mouse, rat, and human oocytes identified that 18%, 12%, and 11% of all DNA methylation, respectively, is linked to transcription initiated at LTRs [78]. Remarkably, LTR-dependent DNA methylation, which in mouse oocytes coincides with transcription-coupled H3K36me3 deposition [79], shows strong species specificity and can be inherited by blastocyst or extraembryonic tissues [78].

### Histone modifications

Post-translational histone modifications (found at promoters or gene bodies) represent a much more complex transcriptional control system, combining more than seventy biochemical modification sites and a few hundred proteins catalyzing the addition or removal of these modifications. Histones are proteins associated with DNA that allow DNA compaction and three-dimensional chromatin organization (structural unit of chromatin: the nucleosome, composed of an octamer of histones [4 histones in two copies each H2A, H2B, H3, and H4] on which 147 bp of DNA is wound). These post-translational enzymatic changes occur in the terminal tails of the histones. The major histone tail modifications are acetylation and methylation, but other potential biochemical modifications exist such as ubiquitination and phosphorylation on specific histone amino acids (lysine can be acetylated, methylated, or ubiquitinated; arginine can be methylated, and serine and threonine can be phosphorylated). Each combination of biochemical modifications (type, position, and number of modifications on histone amino acids) is associated with a particular state of chromatin compaction and thus correlates with different biological effects (transcriptional repression or activation) [80, 81].

Basically, when DNA is methylated, lysine 9 of histone H3 is di- or tri-methylated (H3K9me2/me3), chromatin is compacted, and transcription is blocked. On the contrary, when DNA is demethylated, lysine 9 of histone H3 is acetylated and lysine 4 of histone H3 is di- or tri-methylated (H3K4me2/me3), chromatin is relaxed, and transcription is facilitated.

Much like DNA methylation, post-translational histone modifications are highly dynamic during oogenesis [82] and preimplantation embryo development [83]. For instance, in early-stage growing oocytes, H3K4me3 appears as a canonical pattern at promoters, whereas H3K27me3 appears as a non-canonical form (variant: ncH3K27me3) in regions lacking transcription [84]. Non-canonical H3K4me3 appears at later stages and becomes dominant in mature oocytes. It is broadly deposited in partially methylated domains (which are non-transcribing regions) as H3K27me3 but in non-overlapping subregions [84].

In contrast to oocytes, sperm DNA is mainly packaged by protamines, although it is recognized that a significant proportion of chromatin retains histones which are localized mainly on developmentally important genes [85]. After fertilisation, the spermatic protamines are exchanged for newly synthesized histones derived from the ooplasm [86]. The histone marks (such as H3K4me3 and H3K27me3) seem to be erased more rapidly on the paternal genome than on the maternal genome [87]. The retention of histone marks from the oocyte in the early embryo (two-cell stage) has been evidenced for broad H3K4me3 domains (cover 22% of the oocyte genome) [88]. These large domains become restricted to transcription start sites at embryonic genome activation stage. The active restriction of these broad H3K4me3 domains is required for normal embryo genome activation and further development [89]. In addition, oocyte ncH3K27me3 is specifically removed at developmental gene promoters while oocyte ncH3K27me3 at distal sites persists until the blastocyst stage (inner cell mass) [90]. On the other hand, H3K4me3 and H3K27me3 from sperm (mostly in canonical form) are erased after fertilization, re-established during early development in low levels at broad domains, and then removed at the two-cell (H3K4me3) or epiblast (H3K27me3) stages [88, 90]. Therefore, many of the histone marks inherited in embryos are from the maternal genome and can have important functions during early development.

Histone modifications have also recently been recognized as playing a key role in the regulation of TEs. Transcriptionally, silent TEs are often associated with repressive histone lysine methylation marks (H3K9, H3K27, and H4K20) and histone H2A.Z; however, different marks are specifically enriched in different TEs and cell types [91].

### Epigenetic information through small RNAs

Aside from epigenetic controls, non-coding RNAs (ncRNAs) also control several levels of gene expression [92] and have important roles in signalling networks and the epigenome [93]. There are two types: small RNAs and long non-coding RNAs (lncRNAs). LncRNAs are longer than the arbitrary limit of 200 nucleotides and do not encode proteins [94]. LncRNAs have been implicated in pluripotency and differentiation, and namely in the x-inactivation process through *Xist* and *Tsix* lncRNAs expressed in oocytes and early embryos [95, 96]. Interestingly, lncRNAs expression is also tightly linked with retrotransposons [97]. Small RNAs include microRNAs (miRNAs), small interfering RNAs (siRNAs), and piwi-interacting RNAs (piRNAs). The miRNAs have emerged as powerful post-transcriptional regulators in gene expression. With an average of 22 nucleotides, miRNAs mostly interact with the 3′ untranslated region (3′ UTR) of target mRNAs to initiate mRNA degradation and repress translation and transcription [98]. miRNAs, endogenous small interfering RNAs (endo-siRNAs), and piRNAs are the major types of small RNAs found in mammalian oocytes and early embryos [99].

The presence of miRNAs has been confirmed throughout the growth and maturation of mammalian oocytes [100], and they have a critical role in the physiology and developmental competence of mammalian oocytes and embryos. Furthermore, a recent review provided evidence that aberrant miRNA expression in female reproductive cells and embryos is associated with infertility and embryogenesis defects [101].

miRNAs and piRNAs are required for male germline retrotransposon control [38, 102]. For meiosis and retrotransposon silencing in oocytes, a subclass of siRNAs (endogeneous-siRNAs) known to regulate transcripts [103, 104] is necessary [105, 106]. However, an oocyte-specific piRNA family (os-piRNAs) may also be involved in the silencing of TEs, as revealed in human oocytes [107]. The repression of transposable elements is indeed important for oogenesis, seeing as a higher proportion of oocytes from mice expressing elevated levels of retrotransposons undergo apoptosis during meiotic prophase I [108]. However, the small RNAs required for transposon control could be different in non-murine mammals (including humans), and there is a possibility that piRNAs play a more important or essential role in oogenesis [105]. The functions of these small RNAs underline their importance in the oocyte. However, as reported above, they have been largely underestimated and limited to studies of miRNAs.

## Impact of oocyte vitrification on epigenetic regulators

### DNA methylation

**Fig. 2:**
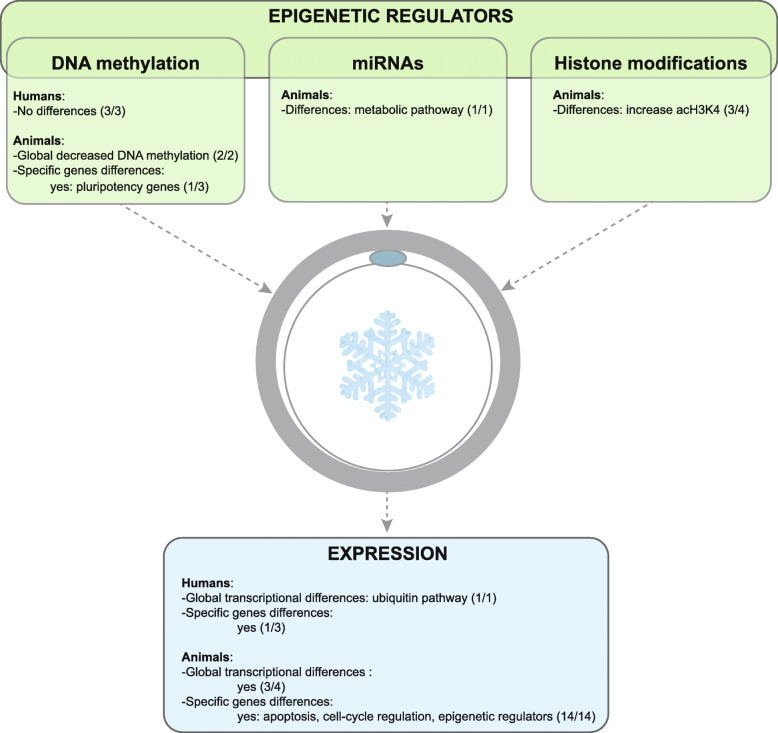
Epigenetic effects of oocyte vitrification in humans and animals (a global overview). Numbers in brackets mean the number of studies reporting differences out of the total number of studies in the literature

Only three studies have been conducted in humans, two of which were conducted using an immunofluorescence (IF) technique, which is a crude way of measuring global DNA methylation state based on the detection of 5mC (Table 1) [109–111]. The third one performed targeted DNA methylation analyses of two selected imprinted genes by using pyrosequencing. One study analyzed the consequences of oocyte vitrification on embryonic methylation levels [110], while the other two studies analyzed the impact of germinative vesicle vitrification on in vitro matured oocytes. In summary, no differences have been reported in humans (Table 1, Fig. 2).

**Table 1:** Impact of oocyte vitrification on DNA methylation in humans

References	Materials	Number of oocytes and embryos	Technology of assessment	Studied sequences	Conclusions
Liu et al. [109]	Vitrified MII, MII from IVM, GV	56 *in vivo* MII, 106 MII after MIV, 122 MII from vitrified GV	Immunofluorescence (5mC)	Global analysis	No significant differences in fluorescence intensities between the three oocyte groups
De Munck et al. [110]	MII from donated oocytes, sibling cohort	31 embryos (D3) from 17 fresh MII and 14 vitrified MII	Immunofluorescence (5mC, 5hmC)	Global analysis	No significant differences in fluorescence intensities between embryos from fresh and vitrified oocytes (5mC 1.0 ± 0.49 vs 0.83 ± 0.41; 5hmC 1.0 ± 0.40 vs 0.81 ± 0.36)
Al-Khtib et al. [111]	MII from IVM (GV donated for research)	77 MII after IVM from 184 vitrified VG, and 85 MII from 120 fresh GV	Pyrosequencing	*H19* (pool of 3 oocytes) and *KCNQ1OT1*	Oocyte vitrification at the GV stage does not affect the methylation profiles of *H19-*DMR et KvDMR1 of the in vitro matured oocytes

*D* day of embryo culture, *GV* oocyte at germinal vesicle stage, *5hmC* 5-hydroxymethylCytosine, *IVM* in vitro maturation, *5mC* 5-methylCytosine, *MII* oocyte at metaphase II stage

In animals, the studies were conducted mainly in cattle and murine animals (Table 2). The studies used various techniques and targeted very different gene categories, so we cannot currently draw strong conclusions relative to the epigenetic impact of oocyte vitrification. Two studies in cattle found a decrease in overall IF methylation after oocyte vitrification [112, 115], while the analysis of three genes subjected to imprinting did not reveal any significant differences [113].

**Table 2:** Impact of oocyte vitrification on DNA methylation and histone modifications in animals

References	Animal model	Materials	Number of oocytes	Number of embryos	Technology of assessment	Studied sequences	Conclusions
Chen et al. [112]	Bovine	Fresh MII, vitrified MII or embryos (D2-D8) from fresh or vitrified MII after IVM	10–15 oocytes or embryos per group		Immunofluorescence	5mC, H3K9ac, H3K9me3	Decrease in the global DNA methylation and H3K9me3 levels and increase in H3K9ac for vitrified MII oocytes. No difference observed specifically in the ICM. Decrease in the level of DNA methylation and H3K9ac in trophectoderm after oocyte vitrification.
Cheng et al. [113]	Murine	Blastocysts (D4) from fresh or vitrified MII		30–45 blastocysts per condition	Bisulfite treatment + sequencing	*H19, Peg3, Snrpn*	No significant differences in oocytes. Decrease in DNA methylation levels for *H19*, *Peg3*, and *Snrpn* in blastocysts after oocyte vitrification.
Zhao et al. [114]	Murine	Fresh MII, vitrified MII	100 oocytes per group		Bisulfite treatment + sequencing	*Dnmt1o*, *Hat1*, promoteur de *Hdac1*	No significant differences.
Hu et al. [115]	Bovine	Fresh MII after IVM, vitrified MII after IVM	150 oocytes per group		Immunofluorescence	Global analysis	Decrease in methylation levels after oocytes slow freezing or after use of DMSO. Increase in methylation levels after using PROH.
Spinaci et al. [116]	Porcine	Fresh MII after IVM, vitrified MII after IVM	H4K5ac 282 fresh oocytes, 192 vitrified oocytes; H3K9me 98 fresh oocytes, 121 vitrified oocytes		Immunofluorescence	H3K9 methylation and H4K5 acetylation	Increase in H4ac level and significant modifications of H3K9me2 levels (decrease or increase) after oocyte vitrification.
Milroy et al. [117]	Murine	Fresh MII, fresh MII after IVM, MII after IVM from vitrified GV	200 oocytes per group		Bisulfite treatment + sequencing	Pluripotency promotors *Oct4*, *Nanog, Foxd3, Sox2*	Increase in the methylation levels of *Oct4* (25%) and *Sox2* (4.5%) promoters after vitrification of in vitro matured oocytes compared to in vivo matured and fresh MII.
Yan et al. [118]	Murine	Fresh MII, vitrified MII	66 fresh, 70 vitrified		Immunofluorescence	H3K9me, H4K5ac	Increase in the H3K9me and H4K5ac levels after oocyte vitrification.
Suo et al. [119]	Murine	Fresh MII, vitrified MII	At least 78 oocytes per group		Immunofluorescence	H4K12ac	Increase in the H4K12ac levels after oocyte vitrification. Zygotes from vitrified MII have disturbed levels before and after appearance of pronuclei.

*D* day of embryo culture, *GV* oocyte at germinal vesicle stage, *ICM* inner cell mass, *IVM in vitro maturation*, *5mC* 5-methylCytosine, *MII* oocyte at metaphase II stage

In an analysis of DNA methylation focused on the enzymes involved in epigenetic changes, Zhao et al. found no differences [114]. Finally, analysis of the methylation levels of pluripotency gene promoters showed that oocyte vitrification did have an effect [117] (Table 2). At the blastocyst stage from vitrified oocytes, a decrease in methylation levels may be observed [112, 113]. This decrease appears to be predominant within the trophectoderm [112].

### Histone modifications

These analyses have only been conducted in animals (Table 2), and the results are complex to interpret because different histone modifications were studied.

However, it seems that oocyte vitrification leads to an increase in the acetylation levels of histones (H4 and H3) [116, 118, 119] though it should be noted that the opposite was found in the trophectoderm [112]. The reported results concerning the methylation levels of histone H3K9 are also inconsistent (Table 2).

### MicroRNAs

A recent study conducted in mice reported the comparison of miRNA transcriptome in fresh and vitrified oocytes [120]. Twenty-two miRNAs were differentially expressed between the two groups, and most of the target genes regulated by these miRNAs were identified as “metabolic pathway” regulators. Among them, miR-134-5p, miR-210-5p, and miR-21-3p were significantly upregulated, whereas miR-465c-5p was downregulated. The expression of potential target PTEN, regulating cell apoptosis through oxidative stress, was reduced [120].

## Impact of oocyte vitrification on gene expression

In humans, only one study has compared the gene expression profiles of fresh and vitrified non-fertilized human oocytes using a microarray approach [121] (Table 3). The authors observed the downregulation of many genes in the ubiquitination pathway, including members of the ubiquitin-specific peptidase family and subunits of the 26S proteasome.

**Table 3:** Impact of oocyte vitrification on expression in humans

References	Materials	Number of oocytes and embryos	Technology of assessment	Studied sequences	Conclusions
D’Aurora et al. [122]	Supernumerary MII, fresh or vitrified	16 fresh, 16 vitrified	RTqPCR	*DCTN3, DCTN1, 2*, and *6, PLK1*	No significant differences.
Monzo et al. [121]	Unfertilized MII (24–78 h post-fertilization), fresh or vitrified	17 fresh, 36 vitrified	Microarray RTqPCR validation	Global analysis (Affymetrix, HG-U133 Plus2.0) 3 genes (*SLC38a2*, *TXNRD1*, *GJA1*)	Significant differential expression between the non-cryopreserved and vitrified MII oocyte pools (608 genes with 509 down and 99 upregulated). Many genes of the ubiquitination pathway were downregulated.
Chamayou et al. [123]	Supernumerary MII, fresh or vitrified	15 fresh, 15 vitrified	RTqPCR	*NAP1L1*, *TOP1*, *H1F0H1, SMC, SCC3, RAD21, SMC1A, SMC1B, STAG3, REC8, CLTA, MAPK6, CKS2, DPPA3, OCT4, FOXJ2*	Overall decrease in the expression after oocyte vitrification with 63.3% of mRNA content maintained after vitrification.
Di Pietro et al. [124]	Supernumerary MII, fresh or vitrified	10 fresh, 15 vitrified	RTqPCR	*HPRT, GAPDH, CYCLOPHILIN, BMP15, GDF9, FIGLA, OCT4, et TAF4B*	No significant differences.

*MII* oocyte at metaphase II stage, *RT-qPCR* quantitative reverse transcription PCR

Three studies have been conducted on supernumerary mature oocytes and expressional analysis of targeted genes. Two of them highlighted that oocyte vitrification did not modify the expression of the selected genes. One analysed the expression of five genes essential for oocyte development and specific functions [124], and the other one focused on the expression of cytokinesis-related genes—Dynactin pathway and subunits [122]. The third human study reported an overall decrease in transcripts involved in DNA structural organization, chromosomal structure maintenance, mitochondrial energetic pathway, cell cycle regulation, and stem cell markers [123].

In summary, the vitrification process may decrease the level of transcripts associated with some of the oocyte’s developmental competencies (Fig. 2).

In animals, from diverse models, four studies have used global transcriptomic analyses (i.e. RNA-sequencing) [125–128] (Table 4). In a murine model, Gao et al. found no differences between vitrified and fresh oocytes [125]. On the contrary, differentially expressed genes were found in bovine and porcine oocytes [126–128]. In particular, transcription regulation, cell differentiation and mitosis, regulation of actin cytoskeleton, and apoptosis pathways were found to undergo changes as a result of the oocyte vitrification process.

**Table 4:** Impact of oocyte vitrification on expression in animals

References	Animal Model	Materials	Number of oocytes and embryos	Technology of assessment	Studied sequences	Conclusions
Wu et al. [129]	Murine	MI and MII after IVM from fresh and vitrified GV	20–25 per group	RTqPCR	*Mps1, BubR1, Mad1, Mad2*	Expression of spindle assembly checkpoint (SAC)-related genes in GV (*Mad1, BubR1,* and *Mad2*), and MII stages (*Mps1* and *Mad1*) were significantly downregulated after vitrification.
Chen et al. [130]	Murine	fresh and vitrified MII	50 per group	RTqPCR	*Gtl2, Peg10, Sirt1, Peg3, Igf2R, H19, Igf2*	*Gtl2* and *Peg10* were significantly increased, *Peg3, Igf2R*, and *Sirt1* were significantly decreased after vitrification
Jia et al. [127]	Porcine	MII after IVM from vitrified and fresh COC	25 per group	RNAseq RTqPCR validation	Global analysis (Illumina) 21 genes	Significant differential expression between the non-cryopreserved and vitrified oocyte pools (19 upregulated genes and 18 downregulated after vitrification and IVM). No GO enrichment or KEGG pathway was identified.
Huang et al. [126]	Bovine	GV, MII after IVM from vitrified GV	3 fresh GV, 4 vitrified GV, 1 fresh MII, and 2 MII derived from vitrified GV	RNAseq	Global analysis (Illumina)	For GV, 12 upregulated genes and 19 downregulated genes after vitrification. No GO enrichment or KEGG pathway was identified. For MII, 47 upregulated genes and 6 downregulated genes after vitrification. With GO and KEGG analyses, several pathways were identified: transcription regulation, cell differentiation and mitosis, regulation of actin cytoskeleton, and apoptosis.
Ma et al. [131]	Bovine	MII after IVM from fresh GV, MII after IVM from vitrified GV, fresh GV, vitrified GV	15 per group (*3 experiments)	RTqPCR	*CD9, CD81, DNMT1*, and *DNMT3b*	The expression of all analysed genes was downregulated after IVM of vitrified GV when compared to the fresh in vitro matured MII oocytes.
Gao et al. [125]	Murine	MII after IVM from fresh GV, MII after IVM from vitrified GV, fresh MII, vitrified MII	100 per group	RNAseq RTqPCR validation	Global analysis (Illumina) *Atp5e, Atp5o, Ndufb9, Uqcrq, Timm17a,* *Dppa5a, H3f3a, Timm13*, and *Tomm40*	No effect of vitrification on the transcriptome. Differences were reported for IVM.
Wu et al. [132]	Bovine	MII after IVM from vitrified GV (liquid nitrogen-LN or helium-LHe), MII after IVM from fresh GV	120 per group	RTqPCR	*p53, EG5, CDC20*, and *NPM2*	For LN-effet, *p53* and *EG5* were upregulated after vitrification, and *CDC20* was downregulated. For LHe-effect, lower effect on the expression of some related genes compared to LN vitrification.
Wang et al. [128]	Bovine	MII after IVM from vitrified GV, MII after IVM from fresh GV	20 per group	RNAseq RTqPCR validation	Global analysis (Illumina) *CDK2,UCHL3, CALM, VDAC2, DPH6, MED27, DAD1, MED21, NR1H4*, and *HMGN1*	Significant differential expression between the non-cryopreserved and vitrified oocyte pools (12 upregulated genes and 90 downregulated genes). At GO analysis, several enrichments in terms of membrane–bounded organelles, macromolecular complex, and intra-cellular part were found. No KEGG pathway was identified.
Shirazi et al. [133]	Ovine	Fresh MII, vitrified MII, fresh GV, vitrified GV	25 per group	RTqPCR	*STAT3, HAT1, HDAC1, SUV39H1, DNMT1, HMGN3a, SMARCAL1*, and *DNMT3b*	The *HMG3a* and *HDAC1* expression was downregulated after vitrification.
Zhao et al. [27]	Bovine	MII after IVM from vitrified GV, MII after IVM from fresh GV	100 per group	RTqPCR	*BAX and BCL2 l1*	The *BAX* expression was upregulated and the *BCL2* l1 expression was downregulated after vitrification.
Dai et al. [134]	Porcine	Fresh MII, vitrified MII	100 per group	RTqPCR	*DNM1, SOD1, MFN2, BAX,* and *BCL2*	The *DNM1* expression was upregulated, and the *SOD1, MFN2, BAX*, and *BCL2* were downregulated after vitrification.
Spricigo et al. [135]	Bovine	MII after IVM from vitrified COC, MII after IVM from fresh COC	20 per group (*4 experiments)	RTqPCR	*DNMT1, SUV39H1, HDAC2, TP53*, and *CASP3*	No effect of vitrification.
Cheng et al. [113]	Murine	Fresh MII, vitrified MII	100 per group	RTqPCR	*Dnmt1,3a,3b,3 l*	The *DNMTs* expression was significantly reduced after vitrification.
Zhao et al. [114]	Murine	Fresh MII, vitrified MII	200 per group	RTqPCR	*Dnmt1o, Hat1,* and *Hdac1 promoters*	No effect of vitrification on the mRNA expression levels of *Hat1* and *Hdac1*. The *Dnmt1o* expression was significantly reduced after vitrification.
Zhou et al. [136]	Bovine	Fresh MII, vitrified MII	50 per group	RTqPCR	*CD9*	The *CD9* expression was downregulated after vitrification.
Rao et al. [137]	Caprine	MII after IVM from vitrified and fresh COC	60 per group	RTqPCR	*GDF9, BMP15, TGFBR1, BPR2, BCL2, BAX* and *P53*	No significant differences of most of the genes.
Turathum et al. [138]	Canine	MII after IVM from vitrified and fresh COC	200 vitrified, 292 fresh	RTqPCR	*HSP70, Dnmt1, SOD1, BAX,* and *Bcl2*	*Bcl2* expression was increased after vitrification, whereas *BAX* was not expressed in both groups.
Anchamparuthy et al. [139]	Bovine	Fresh GV, MII after IVM from vitrified GV, MII after IVM from fresh GV	25 per group	RT-qPCR	*18S rRNA, Fas, FasL, Bax,* and *Bcl-2*	The *Bax* expression was upregulated after vitrification with the ratio of *Bax:Bcl-2* elevated.
Habibi et al. [140]	Murine	MII after IVM from vitrified GV, MII after IVM from fresh GV	10 per group (*3 experiments)	RT-qPCR	*Mater, Sod1*, and *Hook1*	The *Mater* and *Hook1* expression was downregulated after vitrification.
Succu et al. [141]	Ovine	MII after IVM from vitrified GV, MII after IVM from fresh GV	40 vitrified, 24 fresh	RT-qPCR	*b-actin, H2A.Z histone, Poli A, PAP, HSP90b, P34cdc2, Cyclin B, Na/K-ATPase and Type I cadherin*	Except for the *b-actin* and *H2A.Z* expression, all gene expression was downregulated after vitrification.

*GV* oocyte at germinal vesicle stage, *IVM* in vitro maturation, *KEGG* Kyoto Encyclopedia of Genes and Genomes, *LN* liquid nitrogen; LHe = liquid helium, *MII* oocyte at metaphase II stage, *GO* gene ontology, *RNAseq* RNA sequencing, *RT-qPCR* quantitative reverse transcription PCR

When the analyses focused specifically on genes involved in epigenetic modifications, a significant downregulation of gene expression after vitrification has been consistently reported (Table 4) [113, 114, 129–131, 133]. However, Chen et al. recently reported an upregulation of two imprinted genes that are known to play a crucial role in development (*Gtl2* and *Peg10* ) [130].

In bovine and porcine models focused on apoptosis factors, the pro-apoptotic *BAX* gene expression was mostly upregulated, and the anti-apoptotic *BCL2* gene downregulated after the vitrification of mature oocytes [27, 134, 139] (Table 4). However, three studies of cumulo-oocyte complexes found no differences in apoptosis-related gene expression after vitrification warming [135, 137, 138].

In bovine models, a recent study reported an overexpression of cell division-related gene *Eg5* and apoptosis-related gene *p53* [132], while another study described a decreased expression of *CD9* after vitrification, potentially resulting in lowered fertilization capacity [136]. In mouse oocytes, vitrification reduces the expression of genes involved in early embryo development (i.e., *Mater* gene), the positioning of microtubular structures (i.e., *Hook1* gene), and spindle assembly checkpoint-related genes (i.e., *Mps1 and Mad1* genes) [129, 140]. Similarly in ovine oocytes, decreased expression was reported in a panel of developmentally important genes [141].

## Discussion

In recent years, the quest to improve oocyte cryopreservation protocols has remained central in the field of reproductive medicine, which seeks to provide optimal conditions for survival, development, and good clinical outcomes. To date, although vitrification protocols result in relative high survival rates, they do not necessarily signify developmental competence. Previous reports highlighted a decreased cleavage and blastocyst rates after oocyte vitrification in mouse [142, 143], pig [144], cattle [112], and sheep models [141], as well as reduced maturation rates in vitrified immature human oocytes [145]. Additional studies have reported ultrastructural, biochemical, and molecular changes as a result of oocyte vitrification [146].

Very few studies have assessed DNA methylation in humans, which makes it difficult to draw effective conclusions. In addition, the analyses are based either on techniques with low analytical resolution (e.g., IF), or they focused only a few genes subjected to imprinting or specific genes related to cell functions or developmental competences. However, animal studies have shown that oocyte vitrification may (1) modify DNA methylation profiles globally, (2) induce dynamic changes in miRNA content, and (3) cause biochemical changes in histones (high histone acetylation levels).

Epigenetic modifications regulate gene expression so that global DNA demethylation and histone modifications initiate the activation of transcription. Epigenetic changes reported in warmed oocytes could explain the expression changes found in the literature, namely, a global downregulation of expression (in animal models and in humans) [121, 128]. More particularly, in animal models, the dysregulated genes were found to be involved in epigenetic mechanisms (Dnmt enzymes involved in both de novo and maintenance methylation processes and histone-modifying enzymes), in the cell cycle, and in apoptosis regulation [27, 113, 114, 126–128, 134, 139], while in humans, they were found in the ubiquitination pathway [121]. The inhibition of the machinery degradation through the downregulation of ubiquitination may affect the oocyte proteins content and potentially the developmental abilities.

Now, integrative studies associating transcriptomics and proteomics are needed to decipher the metabolic consequences of these types of expressional modifications.

However, most of the epigenetic and expressional changes were observed from vitrification of immature oocytes (GV stage) followed by in vitro maturation step which is known to decrease subsequent embryo development in several mammals, including humans [127, 133, 147, 148]. Furthermore, the effects of vitrification on epigenetic patterns and expression could vary in a manner dependent on species and gene and may also depend on the genomic regions analysed.

Taken as a whole, the literature suggests that epigenetic and transcriptomic profiles are sensitive to the stress induced by oocyte vitrification. As a consequence of the decreasing amount of the stored maternal RNAs until the genome embryonic activation, potential damage to the biological machinery may contribute to impaired embryonic development potential.

However, there is the remaining crucial question of whether these epigenetic and/or expressional changes have any effect on the long-term fate of vitrified oocytes and subsequent offspring. To date, the reports relative to live birth outcomes after oocyte vitrification are sparse [19, 20, 149, 150], and the populations studied are small and poorly or not controlled. The largest study to compare outcomes in vitrified and fresh oocyte groups (including more than one thousand children born after oocyte vitrification) reported reassuring obstetric and perinatal outcomes [19]. However, information regarding the long-term follow-up of these children has not yet been published.

## Conclusion

This literature review highlights that there is a need to learn more about the regulatory mechanisms potentially affected by the oocyte vitrification-warming process, particularly in humans. In addition, despite the overwhelming number of transposable elements, their importance in gametogenesis and development, and their ability to alter genome function, research on the expression of TEs is totally lacking in the field of ART. However, current investigations are facilitated by new technologies that are able to perform a large-scale analysis of DNA methylation (methylome) and transcription (transcriptome) from a small amount of material (as little as a single cell). Finally, the effect of cryo-variables (e.g., type and concentration of cryoprotectors) on epigenetic status and their possible biological implications need to be more fully assessed in order to improve the safety and efficacy of cryopreservation for its diverse applications.

## Data Availability

Not applicable

## Abbreviations

ART: Assisted reproductive technologies
COC: Cumulus-oocyte complex
CpG: Cytosine-guanine dinucleotide
CGI: CpG island
D: Day of embryo culture
DMRs: Differentially methylated regions
DNMTs: DNA methyl transferases
GO: Gene Ontology
GV: Oocyte at germinal vesicle stage
ICM: Inner cell mass
ICRs: Imprinting control regions
IF: Immunofluorescence
IVM: In vitro maturation
KEGG: Kyoto Encyclopedia of Genes and Genomes
LN: Liquid nitrogen
LHe: Liquid helium
MI: Oocyte at metaphase I stage
MII: Oocyte at metaphase II stage
5mC: 5-MethylCytosine
5hmC: 5-HydroxymethylCytosine
miRNAs: MicroRNAs
PGC: Primordial germ cells
RNAseq: RNA sequencing
RT-qPCR: Quantitative reverse transcription PCR
TE: Transposable element
